# Use of Neuronavigation and Augmented Reality in Transsphenoidal Pituitary Adenoma Surgery

**DOI:** 10.3390/jcm11195590

**Published:** 2022-09-23

**Authors:** Miriam H. A. Bopp, Benjamin Saß, Mirza Pojskić, Felix Corr, Dustin Grimm, André Kemmling, Christopher Nimsky

**Affiliations:** 1 Department of Neurosurgery, University of Marburg, 35043 Marburg, Germany; 2 Marburg Center for Mind, Brain and Behavior (CMBB), 35032 Marburg, Germany; 3 EDU Institute of Higher Education, Villa Bighi, Chaplain’s House, KKR 1320 Kalkara, Malta; 4 Department of Neuroradiology, University of Marburg, 35043 Marburg, Germany

**Keywords:** neuronavigation, augmented reality, AR, pituitary adenoma, transnasal, transsphenoidal, intraoperative computed tomography

## Abstract

The aim of this study was to report on the clinical experience with microscope-based augmented reality (AR) in transsphenoidal surgery compared to the classical microscope-based approach. AR support was established using the head-up displays of the operating microscope, with navigation based on fiducial-/surface- or automatic intraoperative computed tomography (iCT)-based registration. In a consecutive single surgeon series of 165 transsphenoidal procedures, 81 patients underwent surgery without AR support and 84 patients underwent surgery with AR support. AR was integrated straightforwardly within the workflow. ICT-based registration increased AR accuracy significantly (target registration error, TRE, 0.76 ± 0.33 mm) compared to the landmark-based approach (TRE 1.85 ± 1.02 mm). The application of low-dose iCT protocols led to a significant reduction in applied effective dosage being comparable to a single chest radiograph. No major vascular or neurological complications occurred. No difference in surgical time was seen, time to set-up patient registration prolonged intraoperative preparation time on average by twelve minutes (32.33 ± 13.35 vs. 44.13 ± 13.67 min), but seems justifiable by the fact that AR greatly and reliably facilitated surgical orientation and increased surgeon comfort and patient safety, not only in patients who had previous transsphenoidal surgery but also in cases with anatomical variants. Automatic intraoperative imaging-based registration is recommended.

## 1. Introduction

Augmented reality (AR) was first used in neurosurgery in the 1980s and was first described by Kelly et al. [1] and Roberts et al. [2]. Injecting overlays of additional information in the optical image of the operating microscope thereby formed the basis for the development of new neurosurgical AR devices and finally commercialization of head-up display (HUD) microscopes in the 1990s, introducing microscope-based AR to the broad neurosurgical community [3,4]. Initially, AR was most often applied in cranial neurosurgery using neuronavigation and thus allowing for a real-time AR visualization of additional information such as outlined lesions or risk structures [5,6,7,8,9,10,11,12]. With the broader availability of state-of-the-art operating microscopes, this technique was also applied in skull base and transsphenoidal surgery [13,14,15,16,17,18].

The transnasal transsphenoidal approach is a common method for the resection of many pituitary lesions. Supported by lateral fluoroscopy and identification of key landmark structures, this allowed for fast and easy orientation making the transsphenoidal approach a somewhat straightforward procedure. However, due to the limited line of sight, surgical orientation can especially be difficult in case of anatomical variants such as reduced intercarotid artery distance that is more frequently seen in patients with acromegaly [19,20], anatomic variation of the sphenoid sinus [21], or previous transsphenoidal surgery (adhesion, fibrosis, and obscured/distorted/missing surgical landmarks) [21,22]. As recently reported, there is also a high inconsistency and wide variation of anatomical landmarks; it is important to bear this inconsistency in mind when relying only on anatomical landmarks and also to comprehensively plan the procedure before surgery [23].

Even though transsphenoidal surgery is considered to be safe with an overall low risk of morbidity or mortality, in a significant number of cases complications occur. In a broad survey among 3172 neurosurgeons, vascular complications were reported in 1.1%, loss of vision in 1.8%, and the incidence of cerebrospinal fluid (CSF) fistulas in 3.9% of the cases; overall mortality was 0.9% [24]. Laws et al. reported a vascular complications in 24 out of 3061 cases (0.78%); 7 were fatal (0.23%) [25].

Considering those perioperative and postoperative obstacles in case of this minimally invasive transnasal transsphenoidal approach, transsphenoidal surgery seems to be an ideal candidate for the application of AR support. The aim of the present study was therefore to report on the clinical experience with microscope-based AR in transsphenoidal surgery compared to the classical non-navigated microscope-based approach.

## 2. Materials and Methods

Out of a consecutive series of 165 patients with intra-/supra- and/or parasellar lesions who had undergone transnasal transsphenoidal surgery performed by a single surgeon (C. N.) between July 2015 and June 2022, 81 have been operated using a classical non-navigated microscopic technique whereas the remaining 84 patients have been operated using neuronavigation and augmented reality support. All individual patients provided written informed consent. The study was approved by the local ethics committee of the University of Marburg (no. 99/18). Due to its retrospective character, no further ethical approval was needed.

### 2.1. Preoperative Planning

In the case of applying neuronavigation and augmented reality preoperative magnetic resonance imaging (MRI), such as contrast-enhanced T1-weighted MRI data or time-of-flight MRI angiography, computed tomography (CT), or CT angiography data is used. After rigid image registration of all required and available data sets using the image fusion element (Brainlab, Munich, Germany) the lesion was outlined manually using the Smart Brush Element (Brainlab, Munich, Germany). In addition, vascular risk structures such as the carotid arteries were outlined manually (slice-based or threshold-based approach). In most cases, an automatic delineation of the optic nerves, optic tracts, and chiasm was provided using the Anatomical Mapping Element (Brainlab, Munich, Germany), in part being manually reshaped to fit the individual image data.

### 2.2. Patient Positioning and Registration

In the case of the classical approach in supine position, the patient’s head is positioned slightly reclined on a closed head ring cushion. Afterwards, the patient’s right leg is positioned for potential preparation of fascia lata in case of CSF leakage.

In the case of the navigated approach the head is fixed in a metallic head clamp (fiducial-/surface-based registration) or carbon head clamp (intraoperative CT (iCT)-based registration) with three metallic pins in a similar fashion with a patient reference array attached to the head clamp. The patient’s leg is positioned accordingly. Afterwards patient registration is performed. Standard patient registration was performed either by preoperatively placing self-adhesive skin markers on the patient’s forehead and acquisition of 3D CT or MRI data one day before surgery and intraoperatively identifying those artificial landmarks using the navigation pointer or by using the z-touch (Brainlab, Munich, Germany) for surface-based registration. Alternatively, patient registration was performed applying automatic iCT-based registration using a 32-slice mobile CT scanner (AIRO^®^ iCT, Brainlab, Munich, Germany) with three self-adhesive skin markers placed on the patient’s forehead within the scanning range. Details of this setup and workflow have been previously reported [26]. For an approximation of effective radiation dosage (ED) a current ED/dose length product conversion factor of 2.4 µSv/mGy*cm was used [27].

Registration accuracy was performed by calculating a target registration error (TRE) by either using an artificial landmark that was not part of the registration procedure (fiducial-based approach) or using the three additional landmarks attached in case of automatic patient registration.

### 2.3. Augmented Reality

For the application of augmented reality, the head-up displays (HUDs) of the operating microscopes, Pentero/Pentero 900/Kinevo 900 (Zeiss, Oberkochen, Germany), were used with no need for further AR supporting devices (e.g., specific glasses). The operating microscope was tracked in space using an attached registration array. Calibration of the AR visualization was inspected by centering the microscope above the central divot of the patient reference array, showing the spatial alignment of the AR visualization of the reference array and the optical outline, and adjusting the alignment if necessary.

All outlined objects (lesion, chiasm, optic nerves, optic tracts, and carotid arteries) can be visualized using the AR display by superimposing the 3D objects in the operating microscope by the integrated HUDs. Besides AR support, multimodal fused image sets are visualized in parallel in the Cranial Navigation Element (Brainlab, Munich, Germany) on a monitor close to the surgical field. Within the Microscope Element (Brainlab, Munich, Germany) all objects can be visualized (semitransparent or solid mode), e.g., superimposed on the microscope video or within a probe’s eye views of the registered image data.

### 2.4. General Setup

**Figure 1:**
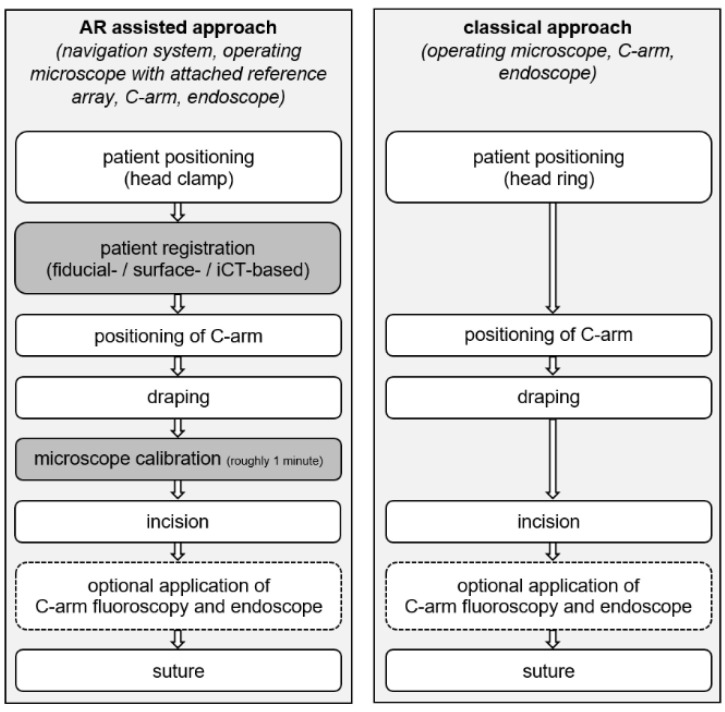
Overall workflow of the AR assisted (**left**) and classical (**right**) approach outlining at which point the additional techniques are integrated in the clinical workflow. Integration encompasses patient registration for navigation purposes as well as calibration of the operating microscope allowing for AR support throughout surgery and is performed prior to incision, not affecting surgery time.

In all cases after C-arm radiographic fluoroscopy was used. In most cases, up to the surgeon’s intraoperative impression, additional endoscope assistance was applied after exposing the sphenoid sinus and opening the sella floor as well as after removal of the tumor. An overview of the overall workflow for the AR assisted and classical approach is provided in Figure 1.

### 2.5. Data Analysis

To compare both technical approaches (traditional vs. navigated and AR assisted) several parameters are investigated:

- Time for intraoperative patient preparation, defined as the duration between beginning of patient positioning and incision.
- Surgery time, defined as the time between incision and suture
- Occurrence of intraoperative CSF leakage followed by reconstruction of dural defects using autologous fascia lata
- Effective dosage of iCT for intraoperative automatic patient registration

## 3. Results

No major complications such as vascular injuries and new neurological deficits were encountered in the recent study cohort. Major indications for the usage of neuronavigation and AR support were in general previous transsphenoidal surgery, but also anatomical variants (e.g., kissing carotid arteries), incomplete pneumatization of the sphenoid sinus, or invasive tumors.

In the study group undergoing surgery using the classical non-navigated microscopic technique (*n* = 81, mean age: 55.19 ± 19.24 years; male/female ratio: 42/39) none of them had previous transsphenoidal surgery. In 66 cases (81.48%) endoscope assistance was used. Intraoperative CSF leakage (small to major) was identified in 35 cases (43.21%) followed by reconstruction using autologous fascia lata. The time for intraoperative patient preparation was 32.33 ± 13.35 min, surgery time was 71.28 ± 29.52 min. In five cases (6.17%) a CSF fistula was seen postoperatively requiring surgical revision, see Table 1.

In the study group undergoing surgery using neuronavigation and AR support (*n* = 84, mean age: 55.95 ± 17.65 years; male/female ratio: 41/43), 18 patients had previous transsphenoidal surgery. In 63 cases (75.00%) endoscope assistance was applied. Intraoperative CSF leakage (small to major) was observed in 36 cases (42.86%) followed by reconstruction using autologous facia lata. The time for intraoperative patient preparation was 44.13 ± 13.67 min. In further detail, patient preparation took 43.40 ± 14.03 min if fiducial-based registration was used, using surface-based 46.33 ± 10.97 min and applying iCT-based registration 44.65 ± 13.54 min were required. Surgery time was 69.87 ± 24.71 min. In three cases (3.57%) a CSF fistula was seen postoperatively requiring surgical revision, see Table 1.

iCT-based registration revealed a target registration error of 0.76 ± 0.33 mm ranging from 0.21 mm to 2.07 mm, whereas fiducial-based registration led to a mean target registration error of 1.85 ± 1.02 mm ranging from 0.55 to 3.43 mm, showing significantly (*p* = 0.001) improved registration accuracy, see Table 1.

The ED of iCT application was significantly reduced over time by omitting a lateral scout scan that was performed in the first cases before CT imaging, by selection of further low dose and finally super-low dose scan protocols, and also by selection of a limited scanning range of 6.2 cm, which was sufficient for patient registration and image fusion with preoperative data. In this way, ED was reduced from 2.566 mSv to 0.041 mSv without any impact on registration quality. Mean ED was 0.128 ± 0.361 mSv, see Table 1.

**Figure 2:**
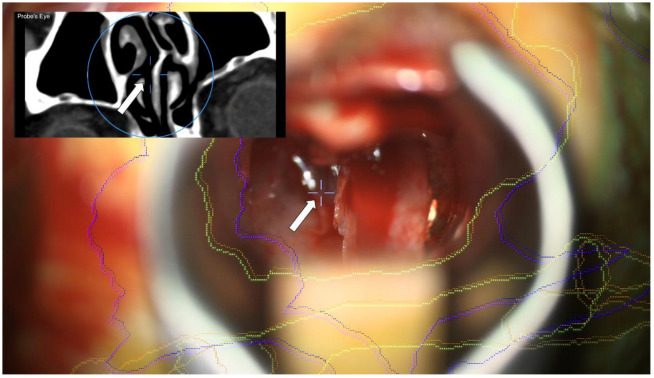
Usage of intraoperative landmarks, in this case, the septum, to evaluate navigational accuracy using the microscope and CT probe’s eye view (left upper corner), showing high navigation accuracy during surgery. Crosshairs (white arrows) showing the focus point in the microscope video and the corresponding CT probe’s eye view (left upper corner).

The overall clinical accuracy of AR application depends on the patient registration accuracy and the microscope registration accuracy. This was on the one hand evaluated by focusing on the patient reference array’s marks and in parallel superimposing the outlines of the reference array. If necessary, the AR visualization was shifted accordingly to improve microscope registration accuracy and thereby also improve overall clinical accuracy. The patient registration accuracy is evaluated using the TRE. In addition to this pre-incision navigational accuracy check, also intraoperative landmarks, if clearly identifiable (Figure 2) can be used to evaluate and thereby ensure continuous high navigational accuracy.

The application of AR clearly enhanced the intraoperative orientation instantly in all cases. Especially in cases with missing clearly identifiable intraoperative landmarks due to previous surgery or patients with anatomical variants, the application of AR support contributed to patient safety and also increased the surgeon’s comfort. The opportunity to, on the one hand, have all relevant objects outlined on preoperative imaging data on a screen close to the surgical field allows for image-based navigation but also to, on the other hand, have various possibilities to visualize the relevant objects directly in the operating microscope using the HUD allowed for a better orientation and a straight-forward understanding of spatial relationships of all relevant structures. Nevertheless, at some point, massive AR information visualized within the microscopic view can also conceal the clear view on the surgical field. If required, the surgeon could decide on temporality switching off selected objects or the HUD completely. They could still use an AR display on the video screen and the navigation screen provide alongside AR support.

### Illustrative Cases

**Figure 3:**
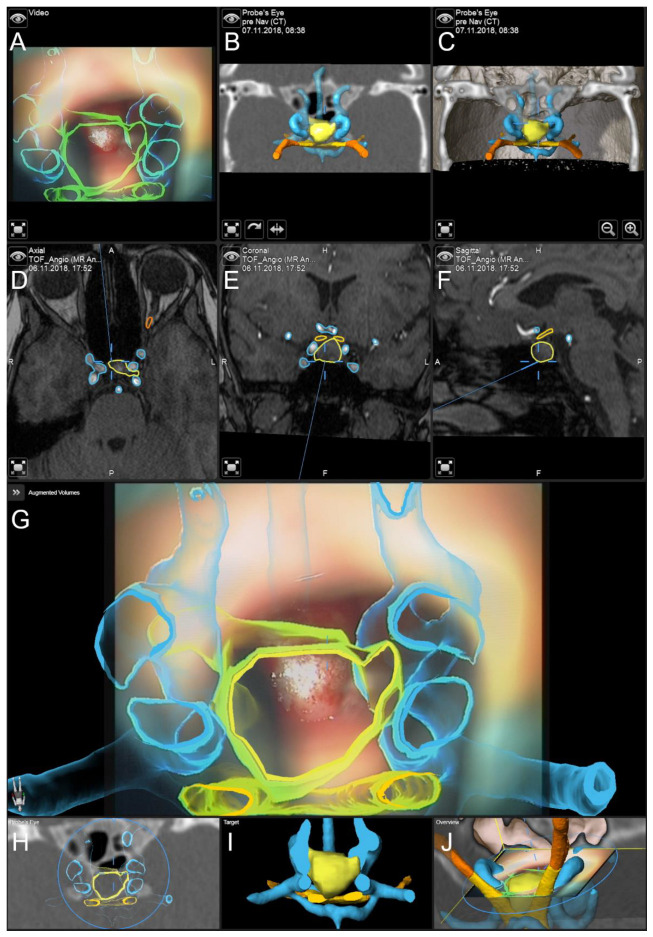
Navigation and AR support in the case of a 37-year-old female patient with a gonadotropic pituitary adenoma. Pre-segmented objects include the lesion (yellow), the carotid arteries (blue), the chiasm (yellow), and the optic nerves (orange). (**A**) Microscope video with 3D visualization of segmented objects using the head-up display. (**B**) The 2D and (**C**) 3D probe’s eye view of intraoperative CT data with 3D visualization of segmented objects. (**D**) Axial, (**E**) coronal, and (**F**) sagittal view (standard navigation) of preoperative time-of-flight MR angiography data with focus on the sella floor. (**G**) AR visualization superimposed on the microscope video with a 3D representation of segmented structures. (**H**) Corresponding probe’s eye view, (**I**) target view (visualizing the lesion (selected as target) in an uncut manner while only parts of the remaining objects distal to the focus plane are displayed), and (**J**) 2D overview depicting the video plane in relation to the 3D visualization of all objects.

In Figure 3, the application of AR support and navigation is presented in a case of a 37-year-old female patient with multiple endocrine neoplasia type 1. Diagnostic follow-up showed a progress of an intra- and suprasellar lesion over the last 14 years since the initial diagnosis. The endocrinological evaluation showed no relevant hormonal insufficiency, vision was not impaired. AR support and navigation allowed for improved intuitive surgical orientation in case of reduced intercarotid distance as seen in this case lowering the risk of vascular complications. The lesion was removed completely without any new endocrinological or neurological deficit. Histopathological evaluation revealed a gonadotropic pituitary adenoma.

**Figure 4:**
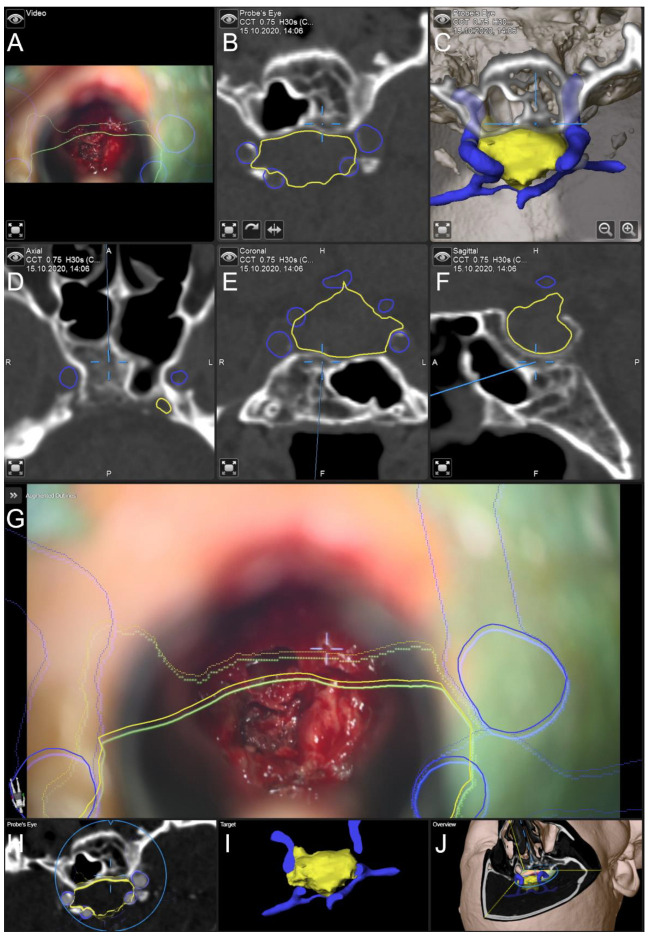
Navigation and AR support in the case of an 84-year-old male patient with previous surgery of a gonadotropic pituitary adenoma. Pre-segmented objects include the lesion (yellow), and the carotid arteries (blue). (**A**) Microscope video with 3D visualization of segmented objects using the head-up display. (**B**) The 2D and (**C**) 3D probe’s eye view of preoperative CT data with 3D visualization of segmented objects. (**D**) Axial, (**E**) coronal, and (**F**) sagittal view (standard navigation) of preoperative CT data with focus on a calcified second layer beyond the sella turcica. (**G**) AR visualization superimposed on the microscope video with a 3D representation of segmented structures. (**H**) Corresponding probe’s eye view, (**I**) target view (visualizing the lesion (selected as target) in an uncut manner while only parts of the remaining objects distal to the focus plane are displayed), and (**J**) 2D overview depicting the video plane in relation to the 3D visualization of all objects.

In Figure 4, the application of AR support and navigation is illustrated in a case of previous surgery. The 84-year-old male patient was clinically admitted with acute headache, nausea, double vision, and underwent surgery for a pituitary adenoma about 20 years before. Diagnostic CT imaging showed an intra- and suprasellar lesion. The endocrinological evaluation suggested complete hormone substitution. The AR support allowed for a straightforward approach to the sphenoid sinus. After opening the sphenoid sinus, a noticeable calcified second layer beyond the sella turcia was identified, in relation to the navigation display. The AR support and navigation allowed for a good orientation with different on-site views enhancing 3D understanding. The soft part of the lesion was removed completely without new endocrinological or neurological deficits. The histopathological evaluation confirmed a gonadotropic pituitary adenoma with increased proliferation. During follow-up, the patient reported no headaches or nausea, subjectively double vision remained.

## 4. Discussion

Comparing the AR supported and classical approach, overall surgical time and complication rates did not differ significantly between both study groups. Whereas preparation time was significantly longer (roughly twelve minutes) in case of AR support in order to set up patient registration (fiducial-/surface- or automatic iCT-based approach), an overall benefit of AR support was seen by easing the surgical orientation not only in patients who had undergone previous transsphenoidal surgery or with missing clearly identifiable landmarks in situ, but also in all other cases and supporting the training of less-experienced surgeons not yet familiar with orientation and surgical strategies within the limited surgical field of view.

Commonly used standard neuronavigation is implemented as a separate navigation display close to the surgical field with the need of using a dedicated navigation instrument (e.g., pointer) and thereof switching surgical instruments and alternating viewing directions throughout surgery for navigation support [28]. To eliminate switching to the navigational pointer during surgery, e.g., the suction tube has been tracked by applying an electromagnetic navigation system in transnasal transsphenoidal surgery [29,30,31] even though unwieldy in the limited area provided by the speculum in the transsphenoidal approach.

A recent study used a navigation probe in endoscopic transsphenoidal surgery to identify, localize and visualize preoperatively outlined neural and vascular structures, showing a match of preoperative segmentation and intraoperative endoscopic and micro-Doppler findings. The authors state that the 3D visualization is highly informative, reassuring experienced surgeons, and could especially also assist less-experienced surgeons to avoid neural of vascular injuries during transsphenoidal surgery [32]. The same was seen in this study with AR supported visualization of lesion outlines and risk structures (e.g., carotid arteries, chiasm, and optic nerves) with no encountered vascular or neurological complications.

Going along with the obstacles of standard navigation using only navigation displays close to the surgical field (interruption of surgery for switching to a dedicated navigation instrument for navigational purposes, alternating viewing directions between surgical site and navigation displays), there was already an early need for virtualizing the physical tooltip by using the microscope’s focal point as an integrated pointer and including all relevant information in the surgical field of view to support the surgeon with transferring relevant information from image space to the real surgical field and to further optimize surgeon comfort and reduce the demand for attention shifts [28,33,34]. The AR support allows for the integration of clinically relevant information in the surgical field of view and thereby also enhances the surgeon’s mental visualization gained from navigation data. Early implementations superimposed object outlines (dashed lines) in the recent focal plane of the operation microscope, perpendicular to the viewing axis, using the microscope’s HUD [11,16,17,18]. The dashed lines outlining the perimeter of target structures two-dimensionally (2D) and the fact that the virtual and real components of the AR scene do not fully match, hamper the depth perception, which is crucial for various surgical tasks such as identification of small/deep targets and avoiding critical close-by structures such as in case of skull base lesions in close relation to neurovascular structures [35,36].

State-of-the-art implementations as presented in this study allow for an improved three-dimensional (3D) perception. Improved resolution of the HUD, color injection to discriminate different objects, smooth real-time visualization provided by the massively increase computing power and further efficient algorithmic implementation facilitate further intuitive use of AR in neurosurgical applications also in combination with 2D and 3D visualization options close-by on the navigation displays. To avoid crowding of the surgical view [33], and to adapt to the individual surgeon’s needs [28] and recent surgical situation the extent, such as the amount and selection of visualized objects, and complexity (2D/3D visualization) can be adjusted at any time during surgery. However, to provide the surgeon with context information outside of the currently visualized sectional planes, in-parallel standard navigation can be considered [13,28].

To rely on AR throughout surgery, especially when performing surgery close to vascular risk structures within a restricted space such as in transnasal transsphenoidal surgery with a limited line of sight, high navigational accuracy is a prerequisite. Standard fiducial- or landmark-based registration approaches as most commonly used [37] are heavily user dependent including the placement of artificial markers (location and amount of markers) [38] before imaging and the intraoperative acquisition of those landmarks using the pointer (e.g., skin shift [39]). In this study TRE (1.85 ± 1.02 mm) for fiducial-based registration ranged from 0.55 mm to 3.43 mm, which is comparable to previous studies of our group [13,40]. Showing this wide range, on the one hand, and the comparably low registration accuracy, on the other hand, documentation of the TRE right after patient registration and in the course of surgery for example using repetitive landmark checks (artificial or anatomical, clearly identifiable in pre- or intraoperative imaging data) is mandatory to monitor overall clinical and AR accuracy. To overcome especially low registration accuracy due to the user-dependent registration procedure, intraoperative automatic patient registration procedures such as iCT-based registration reveal a significantly lower mean TRE of 0.76 ± 0.33 in this study is therefore highly recommended, as without sufficient high clinical accuracy the AR display might give a false sense of security. Besides navigational accuracy, the AR accuracy can also be finely tuned using the navigation update feature provided in the microscope application by superimposing a semitransparent minimum intensity projection (MIP) of bony structures reconstructed out of CT imaging data or object outlines in the recent microscope’s focus plane. By adjusting the MIP/object visualization using in-plane translation and rotation, spatial navigation accuracy can be improved as reported for cranial surgery applying comparable cortical vessel alignment [41].

In case of inaccurate patient registration, positional shifting of the patient’s head in relation to the attached patient reference array, or loss of accuracy throughout surgery (e.g., effects of draping [42]), AR support provides an additional principle advantage over purely standard pointer-based navigation. The size and spatial relation of outline objects remains correct even though spatially transformed. In case this transformation can be estimated using intraoperative landmarks, it can also be estimated for all objects, allowing for at least a rough orientation. Nevertheless, if highly reliable navigation is required, in case of intraoperative events, a re-registration, e.g., using a repeated low-dose iCT-based registration scan, allows for a reestablishment of high navigational accuracy [13].

The use of super low-dose iCT protocols for automatic iCT-based patient registration allowed for a tremendous reduction in the effective radiation dose from 2.566 mSv to 0.041 mSv (1.60%) and is thereof in a range of a single chest radiography. As demonstrated in a recent study reduction in radiation dose from high-dose to low-dose protocols in this setup did not diminish overall patient registration accuracy [26]. An improved registration accuracy with a mean error of 1.28 mm was seen in the application of intraoperative cone beam CT for registration of an endoscopic video navigation system compared to a tracker localization error of about 1–2 mm also demonstrating the opportunity to drastically reduce the applied dosage to roughly 1/100th of the dose of a typical diagnostic CT of the head [43].

The use of AR support and thereof navigation support might require additional preoperative and intraoperative procedural time. However, studies on the extent and significance of time reduction/time increase are rare [22]. As also in the classical approach a thorough planning of the surgical procedure is highly recommended [23] also preoperative segmentation is somehow time-consuming. One study reported a required planning time of ten minutes for a transsphenoidal approach with sufficient image data quality [44]. Due to the retrospective analysis in this study, preoperative planning time was not acquired in all cases, but given the diverse planning tool offered by the used navigation system, preoperative planning of transsphenoidal procedures outlining the lesion and vascular and neural risk structures is available in a moderate amount of time, depending on image quality and clinical and technical user experience. For overall operative time, mixed effects are seen [22]. Most set-ups analyzed in a recent review include different platforms, microscope-based or endoscope-based AR systems, and differing definitions of “used time” (e.g., overall and surgical) making results hard to interpret. In this study, no significant difference in surgical time (incision to suture) is seen in both groups, whereas preparation time (beginning to patient positioning to incision) in one group including the additional requirement of patient registration differed significantly, the same accounts for overall time incorporating patient preparation and surgical time. However, in line with [22], prolonged time might be more dependent on the familiarity of the whole team (OR staff, technical staff, anesthesia team, and surgeons) with the technology, even though a mean additional time in the pre-surgical phase for patient registration of roughly twelve minutes as in this study seems reasonable. Nevertheless, further studies are needed to evaluate the effect of AR support on pre- and intraoperative time.

Intraoperative CSF leakage frequently occurs in transsphenoidal surgery of pituitary lesions with reported rates of over 50% with various grades ranging from small leaks without obvious diaphragmatic defect, to large diaphragmatic or dural defects [45,46]. In this study intraoperative CSF leakage was seen in 43.21% (classical approach) and 42.86% (AR supported approach), not differing significantly. The rate of postoperative CSF fistula also did not differ significantly among both groups (6.17% vs. 3.57%), being in a comparable range of reported postoperative CSF fistulas (3.90%), slightly higher in the study cohort using the classical non-AR supported approach [24].

The integration of endoscopes, not only used in addition to the microscope-based transsphenoidal approach or as a standalone tool in endoscopic transsphenoidal surgery, into the navigation system enables the visualization of the endoscope’s tip in relation to anatomical structures also serving as a navigational “pointer” [30]. Concerning the benefits of AR support in the microscope-based set-up also the endoscope-based set-up can benefit from the integration of AR support to improve orientation and ease identification of target and especially risk structures. The optically distorted geometry of the endoscope video might thereby be an obstacle to address when AR support is implemented. First implementations of AR support in endoscope-based surgery have been demonstrated already in 2002 [47], continuously improved with a further broadened field of view [48], used in combination with robotic devices [49], e.g., for integration of virtual endoscopy [50].

AR support can also assist in education and training of residents and less-experienced surgeons allowing for training and practice on the one hand outside of the OR but also on the other hand during surgery [8] providing especially in case of transsphenoidal approaches an improved and eased surgical orientation also in “straightforward” cases, allowing for a mental mapping of surgical trajectory, surgical field, intraoperative landmarks, and image data.

One limitation of this study is its retrospective nature, as especially time parameters are dependent on the surgical routine documentation, not separating all phases of surgery as possibly required within this study (e.g., time for planning or explicit time for patient registration is not documented). Especially in cases of previous transsphenoidal surgery or patients with anatomic variants, a control group of patients undergoing surgery without AR support is missing. Due to the low reported numbers of severe complications of smaller than 1–2% [24], depending on the surgeon’s experience, a comparative study to prove increased patient safety seems unethical (e.g., surgery performed by a rather inexperienced surgeon to encounter complications) or impractical (e.g., large required patients cohort).

## 5. Conclusions

AR support in transsphenoidal surgery eases surgical orientation, especially in cases with anatomical variant or in patients who had undergone previous transsphenoidal surgery with clearly missing identifiable landmarks. Even though slightly more time is needed to set-up patient registration, AR support also seems to enhance orientation in all other cases and, therefore, might also assist in training of less-experienced surgeons who are becoming more familiar with surgical orientation and strategies in the narrow space provided by the speculum in the transsphenoidal approach. However, high patient registration accuracy and overall clinical accuracy, as supported by automatic intraoperative imaging-based registration procedures, is a prerequisite.

## Figures and Tables

**Table 1:** Summary of results.

	Study Cohort (Classical Approach)	Study Cohort (AR Supported)	*p*-Value
number of patients	81	84	-
mean age (years)	55.19 ± 19.24	55.95 ± 17.65	0.792 ^4^
male/female ratio	42/39	41/43	0.696 ^3^
endoscopic assistance	66 (81.48%)	63 (75.00%)	0.314 ^3^
previous surgery	0	17	-
intraoperative CSF leakage	35 (43.21%)	36 (42.86%)	0.964 ^3^
major complications	0	0	-
postoperative CSF fistula	5 (6.17%)	3 (3.57%)	0.437 ^3^
patient preparation time (min)	32.33 ± 13.35	44.13 ± 13.67	<0.001 ^4^
surgery time (min)	71.28 ± 29.52	69.87 ± 24.71	0.739 ^4^
TRE (fiducial) (mm) ^1^	n.a.	1.85 ± 1.02 [0.51; 3.43]	0.001 ^5^
TRE (iCT) (mm) ^1^	n.a.	0.76 ± 0.33 [0.21; 2.07]	
ED (iCT) (mSv) ^2^	n.a.	0.128 ± 0.361 [0.041; 2.556]	-

AR augmented reality, TRE target registration error, iCT intraoperative computed tomography, ^1^ TRE only applicable for fiducial-based and automatic iCT-based registration, ^2^ only applicable for automatic iCT-based registration, ^3^ Chi-Quadrat-Test, ^4^ homogeneity of variances was assessed using Levene’s Test showing homogeneity of variances, therefore a *t*-test is applied, ^5^ homogeneity of variances was assessed using Levene’s Test showing no homogeneity of variances, therefore, a Mann–Whitney-U test was used. The significance level was set to *p* < 0.05.

## Data Availability

The data in this study are available on request from the corresponding author. The data are not publicly available due to privacy restrictions.
